# How do Italian pediatric endocrinologists approach gender incongruence?

**DOI:** 10.1186/s13052-023-01471-2

**Published:** 2023-06-14

**Authors:** Silvia Ciancia, Lorenzo Iughetti

**Affiliations:** Pediatric Unit, Department of Medical and Surgical Sciences of the Mother, Children and Adults, University of Modena and Reggio Emilia, Largo del Pozzo 71, Modena, 41124 Italy

**Keywords:** Gender dysphoria, Gender incongruence, GnRHa, Gender affirming hormones, Puberty

## Abstract

**Background:**

Gender incongruence (GI) is a term used to describe a marked and persistent incompatibility between the sex assigned at birth (SAAB) and the experienced gender. Some persons presenting with GI experience a severe psychological distress defined as gender dysphoria (GD).. Although the prevalence of GI is probably underestimated, recently a great increase in numbers of transgender and gender diverse (TGD) youths presenting at the gender clinics has been registered. After a careful multidisciplinary evaluation and upon acquisition of informed consent from the youth and the legal guardian(s), puberty suppression can be started in TGD youths, followed by the addition of gender affirming hormones (GAH) by the age of 16 years. Although Italian specific guidelines are available, their application is often complex because of (among other reasons) lack of specialized centers and healthcare professional with experience in the field and the regional differences within the Italian healthcare system.

**Main body:**

To investigate the care offered to TGD youths across Italy, we proposed a survey of 20 questions to the directors of the 32 Italian Centers of pediatric endocrinology participating to the Study Group on Growth and Puberty of the Italian Society of Pediatric Endocrinology (ISPED). Eighteen pediatric endocrinologists representative of 16 different centers belonging to 11 different regions responded to the survey. In the large majority of centers TGD youths are taken in charge between the age of 12 and 18 years and at least three healthcare professional are involved. Most of Italian pediatric endocrinologists follow only a very limited number of TGD youths and reference centers for TGD youths are lacking.

**Conclusion:**

There is an urgent need for gender clinics (homogeneously distributed on the national territory) where TGD youths can access high standard care.

**Supplementary Information:**

The online version contains supplementary material available at 10.1186/s13052-023-01471-2.

## Background

Gender incongruence (GI) is a term used to describe a marked and persistent incompatibility between the sex assigned at birth (SAAB) and the experienced gender. Transgender and gender diverse (TGD) persons can feel highly uncomfortable with primary and secondary sexual characteristics and the social role associated to the SAAB and they can experience a severe psychological distress defined gender dysphoria (GD) [1]. The prevalence of GI among TGD youths is difficult to determine, however over the past decades a great increase of the number of TGD youths presenting at the gender services has been registered [2–4].

A recent systematic review estimated the prevalence of GI among children and adolescents between 1.2% and 2.7% (increasing up to 2.5–8.4% when a broader non binary concept of gender diversity is investigated) [5]. Also, at the present youths assigned female at birth (AFAB) represent around 70% of the TGD youth with an inversion of the previously known trend. ) Although Italian guidelines have been published almost 10 years ago [6], a uniform delivery of care to Italian TGD youths has still not been accomplished. Several factors may play a role in this, such as the regional differences within the Italian healthcare system, the insufficient number of trained healthcare professionals, the lack of homogenous distribution of reference centers nationwide (according to the National Observatory on Gender Identity, ONIG, only nine centers provide care to TGD youths, two in the South, five in the center and two in the Nord of Italy).

According to international and national guidelines [6–8], puberty suppression through Gonadotropin-Releasing Hormone agonists (GnRHa) can be prescribed to TGD youths after a diagnosis of marked and persistent GI is made and puberty is progressed at least at Tanner stage 2, upon acquisition of informed consent from the youth and the legal guardian(s). This treatment will delay the development of undesired secondary sexual characteristics and give time to the youth to explore his/her gender identity. By the age of 16 (or a bit earlier in some countries) gender affirming hormones (GAH) can be added if GI is persistent and the adolescent is willing to proceed in the transition. As established by the Italian Medicines Agency (AIFA) in February 2019, all these medications are free of charge for the TGD individuals. In Italy, the TGD individuals (aged > 18 years) willing to proceed with gender affirming surgery will have to require a formal authorization to the Court. Since 2015, gender affirming surgery is no longer required to obtain the change of name and gender. Although gender affirming treatment for TGD youths is still characterized by an ongoing debate, the delay (or the deny) of treatment when necessary, might have extreme consequences on the health of the youth.

## Main text

We proposed a survey of 20 questions to the directors of the 32 Italian Centers of pediatric endocrinology participating to the Study Group on Growth and Puberty of the Italian Society of Pediatric Endocrinology (ISPED) to investigate the approach of the Italian pediatric endocrinologists to GI. The questions of the survey are shown in the supplement.

Eighteen pediatric endocrinologists representative of 16 different centers belonging to 11 different regions (5 in the North, 2 in the center, 2 in the South and 2 islands) responded to the survey.

. Colleagues of 14 centers reported to treat TGD youths, but 64% had in follow-up less than 10 individuals. Only one center reported to have in charge more than 20 TGD youths. According to the experience of 50% of colleagues, GI is more frequent among youths assigned male at birth while according to 36% GI has a higher prevalence in youths assigned female at birth. In 79% of centers TGD individuals receive the diagnosis of GD between 12 and 18 years of age and at least three healthcare professionals are involved (more often a child and adolescent psychiatrist, a psychologist and a pediatrician). In only 3 centers a bioethicist is part of the team. 79% of the interviewees consider appropriate to start puberty suppression only in selected patients and 50% thinks that puberty suppression should be started between 10 and 12 years of age (if puberty is already begun). According to 50%, gender affirming hormones should be started before the age of 16. Mental health disorders (including eating disorders) are often diagnosed in TGD youths and 50% of centers follow TGD youths who take psychotropic medications (mainly antidepressants). 36% of centers include in the follow-up provided to TGD youths several investigations to evaluate the effect of the treatment and possible side effects, such as the evaluation of glucose metabolism, lipid profile, coagulation profile, bone mineralization and mental health assessment. .

Only 21% of centers follow TGD individuals who chose to proceed with gender affirming surgery; however this is most likely due to the fact that surgery is performed after the age of 18, thus after the transition to adult care. Only 12% of interviewees think that surgery should be offered earlier, specifically by the age of 16. In 29% of centers, more than 50% of TGD youths are victims of bullying (according to personal estimations), while in 43% of centers the percentage is lower (10–50%). 29% reports early school drop out in < 30% of cases, 21% reports an incidence > 30% and 21% does not estimate a higher incidence of school drop out in TGD youths. 36% of the interviewees affirm that more than 50% of TGD youths is satisfied with the body image obtained with to the treatment, while only 14% refers a satisfaction rate > 90%.

## Conclusion

Although international and national guidelines for gender affirming treatment in TGD youths are available, not all pediatric endocrinologists are familiar with them or have enough expertise in this specific field. From our survey resulted that most of Italian pediatric endocrinologists offering care to TGD youths follow only a very limited number of individuals while we think that TGD youths should be offered by highly specialized centers where an experienced multidisciplinary team provides care. The care should be homogeneously distributed on the national territory. Our survey has clearly some limitations such as the distribution among a selected study group within ISPED and the lack of involvement of endocrinologists (which in some centers might offer care to both TGD youths and adults). Also we did not investigate in details the adherence to the national guidelines but we aimed to have a general overview on this topic and mainly to sensitize Italian pediatric endocrinologists on the increasing number of TGD youth seeking care and deserving to receive it. We think that the opening of gender clinics with specific focus on youths should be promoted in Italy and uniform protocols of care should be applied.

## Electronic supplementary material

Below is the link to the electronic supplementary material.

Supplementary Material 1

Supplementary Material 2

## Data Availability

Not applicable.
