# Supplementary material for: How do Italian pediatric endocrinologists approach gender incongruence?
Source: Ital J Pediatr. 2023 Jun 14;49:70. doi: 10.1186/s13052-023-01471-2 (PMC10268452; doi:10.1186/s13052-023-01471-2)
Supplement: Supplementary file 1 — Supplementary Material 1 [file 13052_2023_1471_MOESM1_ESM.docx]

| Do you follow transgender gender diverse (TGD) youths?   - No - Yes (please specify how many)   - <10 - 10-20 - 20-30 - 30-40 - >40 | Did some of TGD youths in your center decide to proceed with affirming gender surgery?   - Yes - no |
| --- | --- |
| The sex assigned at birth (SAAB) is:   - Mainly male - Mainly female - Male and female in similar proportion | From what age should it be allowed to offer gender affirming surgery?   - >14 years - 14-16 years - 16-18 years - >18 years |
| Around which age is the gender incongruence (GI) diagnosed?   - <6 years - 6-12 years - 12-18 years - >18 years | Do some of TGD youths in follow-up at your clinic assume psychotropic medications?   - No - Yes - Antidepressants - Benzodiazepines - Antipsychotics - Other (please specify): |
| Which healthcare providers are involved in the multidisciplinary team?   - Child and adolescent psychiatrist - Psychiatrist - Psychologist - Pediatrician - Endocrinologist - Other (please specify): | Which percentage of TGD youths in follow-up at your clinic are satisfied with the results of the gender affirming treatment on the body image?   - <20% - 20-50% - 50-70% - 70-90% - >90% |
| Which healthcare providers are primary involved in the communication with the family and youth?   - Child and adolescent psychiatrist - Psychiatrist - Psychologist - Pediatrician - Endocrinologist - Other (please specify): | How many TGD youths in follow-up at your clinic are victims of bullying?   - <10% - 10-50% - >50% |
| Do you agree with the administration of GnRHa in order to block the further development of secondary sexual characteristics?   - Yes - No - Only in some cases | How many TGD youths in follow-up at your clinic drop out school?   - none - 10-20% - 20-30% - 40-50% - >50 |
| At what age do you think it is more appropriate to start the administration of GnRHa (if puberty started)?   - <10 years - 10-12 years - >12 years - Other (please specify): | How many years of experience in pediatric endocrinology do you have?   - <5 - 5-10 - 10-20 - >20 |
| At what age do you think gender affirming hormones (GAH) should be administered?   - <10 years - 10-12 years - 12-14 years - 14-16 years - >16 years | In which Italian region do you work? |
| Do (some) TGD youths suffer of medical conditions?   - No - Yes - Mental health disorders including eating disorders - Impaired bone mineralization - Cardiovascular disorders - Other (please specify): | Are you ?   - Female - Male - Other |
| Which investigations do you perform during the follow-up?   - Assessment of the glucose profile - Assessment of the lipid profile - Assessment of the coagulation - DXA - Other (please specify): | Do you work in a   - General public hospital - Univeristy hospital - Private hospital |
