# Supplementary material for: How do Italian pediatric endocrinologists approach gender incongruence?
Source: Ital J Pediatr. 2023 Jun 14;49:70. doi: 10.1186/s13052-023-01471-2 (PMC10268452; doi:10.1186/s13052-023-01471-2)
Supplement: Supplementary file 2 — Supplementary Material 2 [file 13052_2023_1471_MOESM2_ESM.doc]

30 November 2026

Dear Editor,

We are sending to your attention the revised manuscript titled *“***How do Italian pediatric endocrinologists approach gender incongruence?”** for the purpose of a possible publication as Commentary in your prestigious journal.

Thank you for your judgments on the scientific aspects of our paper and for considering it interesting.

Gender incongruence and consequent gender dysphoria are increasing in childhood and adolescence. This condition requires a multi-disciplinary approach with a specific role for pediatric endocrinologists. In this commentary we briefly report the results of a survey between the Italian Centers of pediatric endocrinology focused on their approach to patients with gender dysphoria.

We have followed the reviewers' comments carefully and we are sending a largely revised version of the manuscript. We are attaching a response for reviewers with detailed answers to each single point raised by the reviewer. Text changes are underlined

The revised version of the manuscript has been read and approved by both the authors.

I’m very grateful to you since now for your attention.

Sincerely Your

Lorenzo Iughetti

Lorenzo Iughetti, M.D., Ph.D.

Department of Pediatrics

University of modena & reggio emilia

Via del pozzo, 71 - 41100 Modena, Italy

Tel. +39 059 4222182 Fax. +39 059 4224583

e-mail: [iughetti.lorenzo@unimore.it](mailto:iughetti.lorenzo@unimore.it)
